# Genome-Wide Association Analysis Reveals Genetic Loci and Candidate Genes for Chest, Abdominal, and Waist Circumferences in Two Duroc Pig Populations

**DOI:** 10.3389/fvets.2021.807003

**Published:** 2022-01-28

**Authors:** Shenping Zhou, Rongrong Ding, Zhanwei Zhuang, Haiyu Zeng, Shuxian Wen, Donglin Ruan, Jie Wu, Yibin Qiu, Enqin Zheng, Gengyuan Cai, Jie Yang, Zhenfang Wu, Ming Yang

**Affiliations:** ^1^College of Animal Science and Technology, Zhongkai University of Agriculture and Engineering, Guangzhou, China; ^2^College of Animal Science and National Engineering Research Center for Breeding Swine Industry, South China Agricultural University, Guangzhou, China; ^3^Guangdong Wens Breeding Swine Technology Co., Ltd., Yunfu, China

**Keywords:** single-trait GWAS, Duroc pigs, chest circumference, abdominal circumference, waist circumference, multi-trait GWAS

## Abstract

Chest circumference (CC), abdominal circumference (AC), and waist circumference (WC) are regarded as important indicators for improving economic traits because they can reflect the growth and physiological status in pigs. However, the genetic architecture of CC, AC, and WC is still elusive. Here, we performed single-trait and multi-trait genome-wide association studies (GWASs) for CC, AC, and WC in 2,206 American origin Duroc (AOD) and 2,082 Canadian origin Duroc (COD) pigs. As a result, one novel quantitative trait locus (QTL) on *Sus scrofa* chromosome (SSC) one was associated with CC and AC in COD pigs, which spans 6.92 Mb (from 170.06 to 176.98 Mb). Moreover, multi-trait GWAS identified 21 significant SNPs associated with the three conformation traits, indicating the multi-trait GWAS is a powerful statistical approach that uncovers pleiotropic locus. Finally, the three candidate genes (*ITGA11, TLE3*, and *GALC*) were selected that may play a role in the conformation traits. Further bioinformatics analysis indicated that the candidate genes for the three conformation traits mainly participated in sphingolipid metabolism and lysosome pathways. For all we know, this study was the first GWAS for WC in pigs. In general, our findings further reveal the genetic architecture of CC, AC, and WC, which may offer a useful reference for improving the conformation traits in pigs.

## Introduction

Due to conformation traits closely related to many economic traits in livestock, these traits are considered as an important breeding selection criteria. For instance, Ohnishi and Satoh (1) reported a positive genetic correlation between chest circumference (CC) and backfat thickness (0.6) in Duroc pigs. Moreover, CC was also closely associated (*r* > 0.7) with body weight in pigs (2). Vargas et al. (3) found a linear relationship between live weight and CC and abdominal circumference (AC) in goats. Although there were almost no waist circumference (WC) studies in livestock, the WC was reported to predict non-abdominal, abdominal subcutaneous, and visceral fat in humans (4). Thus, understanding the genetic architecture of CC, AC, and WC will help improve the economic traits related to these conformation traits in livestock.

In recent years, with the rapid development of sequencing technology and dense marker panels, genome-wide association study (GWAS) has become a reliable approach that detects genetic variants associated with the trait of interest. It has become widely used in humans (5), plants (6), and animals (7). However, all significant single nucleotide polymorphisms (SNPs) together only explained a proportion of heritability in many GWAS studies (8, 9). The main reason is that most complex traits are affected by many small-effect SNPs, and the sample size is too small to identify these small-effect SNPs (5, 7). In addition, these small-effect SNPs collectively account for most of the heritability of complex traits (10). Although that increasing sample size can enhance statistical power in GWAS studies, it is too expensive. Thus, most GWAS for the complex traits are conducted based on the limited sample size of a single population, leading to limited statistical power. When the sample size is insufficient, it is an effective method to use a combination strategy to increase the power to detect associations, such as single-trait and multi-trait GWAS (11, 12). The clear advantage of multi-trait GWAS is that it can detect interactions and pleiotropic loci, but the statistical power of this analysis is affected by the correlation between traits (13). In short, the multi-trait GWAS can complement the single-trait GWAS results, and thereby the combination of both can increase the statistical power of GWAS.

Few GWAS research on the conformation traits in pigs, especially the CC, AC, and WC traits. To date, 2,492 quantitative trait locus (QTLs) are associated with conformation traits, including 21 and four QTLs are associated with CC and AC in the pig QTL database (https://www.animalgenome.org/cgi-bin/QTLdb/SS/index, February 04, 2021). Notably, no QTLs are associated with WC in the pigQTLdb. Furthermore, only a small part of these QTLs were detected by GWAS. Here, we conducted single-trait and multi-trait GWASs for CC, AC, and WC on 2,206 AOD pigs and 2,082 COD pigs. This study aimed to identify more loci associated with CC, AC, and WC traits using single-trait and multi-trait GWASs, further understand the genetic architecture of the three traits in pigs.

## Materials and Methods

### Animals and Phenotypic Data

**Figure 1:**
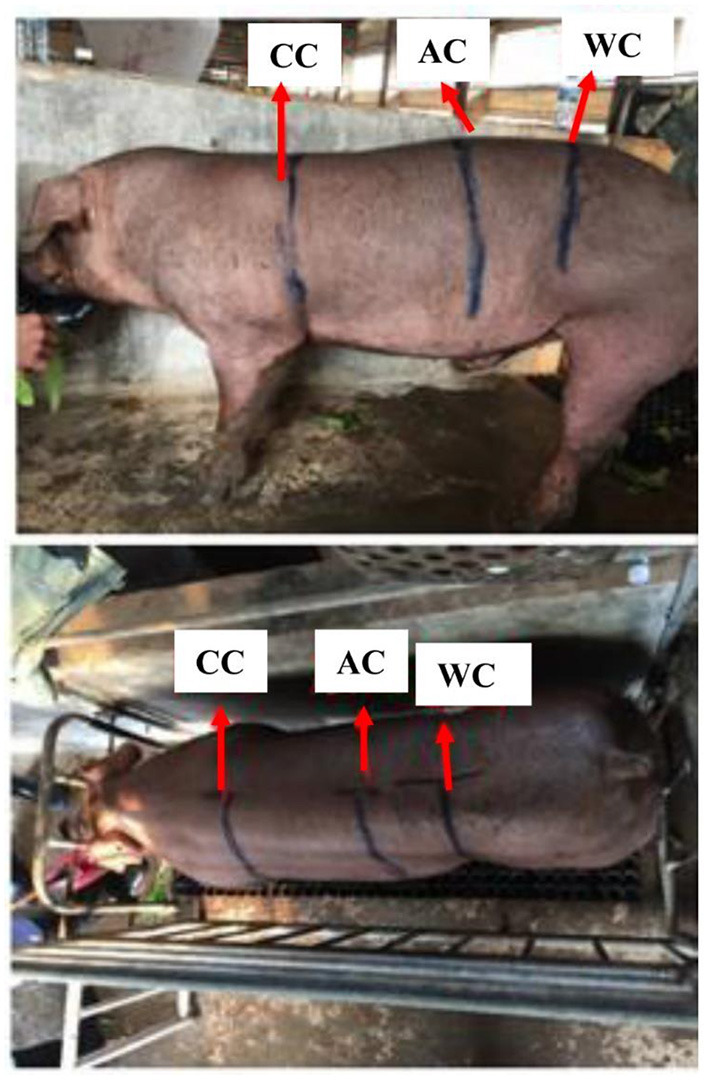
The measuring parts of CC, AC, and WC in pigs. CC, chest circumference; AC, abdominal circumference; WC, waist circumference.

From 2013 to 2017, a total of 2,206 AOD pigs (*N*_*male*_ = 718, *N*_*female*_ = 1,488) and 2,082 COD pigs (*N*_*male*_ = 1,010, *N*_*female*_ = 1,072) were collected from the Guangdong Wens Foodstuff Group Co., Ltd. (Guangdong, China). All the animals were reared under the same feeding conditions. When the pigs reached the live weight of 100 ± 15 kg and were fasted for 24 h, their CC, AC, and WC were measured by a tape measure. The measuring positions of CC, AC, and WC were shown in Figure 1. The CC, AC, and WC were measured by circling the trailing edge of the scapula, the largest part of the abdomen, and the front edge region of the hind leg in the pigs, respectively.

### Genotyping and Quality Control

Ear tissue was collected from all animals, and genomic DNA was extracted from ear tissue by the standard phenol-chloroform method. DNA quality was assessed by ratios of light absorption (A260/280 and A260/230) and electrophoresis. Qualified DNA concentration was diluted to 50ng/μl. All animals were genotyped with the GeneSeek Porcine 50K SNP chip (Neogen, Lincoln, NE, United States) that contains 50,703 SNP across the whole genome. The quality control (QC) was performed by PLINK v 1.9 (14). Briefly, individuals with call rates more than 95% and SNPs with call rates more than 99%, minor allele frequency more than 1%, and Hardy-Weinberg *P*-value more than 10^−6^. Moreover, SNPs unmapped regions and located on the sex chromosomes were filtered. Notably, the two Duroc pig populations obey the same QC criteria. After genotype QC, the qualified individuals and SNPs were used for subsequent single-trait and multi-trait GWAS analyses (Supplementary Table 1).

### Population Structure Analysis

Due to population stratification that may cause false-positive findings in GWAS, principal component analysis (PCA) and quantile-quantile (Q-Q) plots were conducted for two Duroc populations to assess and correct population structure. In this study, PCA and Q-Q plots were generated by software GCTA v1.92.4beta (15) and R v3.6.1 software (16), respectively.

### Single-Trait GWAS

GEMMA v0.98 software was used to conduct a univariate linear mixed model (LMM) to detect the association between each SNP and phenotype (17). The statistical model is described as follows:


y = Wα + Xβ + u +ε

where *y* denotes a vector of phenotypic values; *W* refers to the incidence matrices of covariates (fixed effects), including the top five eigenvectors of PCA, sex, and live weight; α is the vector of corresponding coefficients with the intercept; *X* represents the vector of SNP genotypes; β stands for the corresponding effect size of the SNP; *u* is the vector of random effects with *u* ~ MVN_n_(0, λ τ^−1^K); ε corresponds to the vector of random residuals with ε ~ MVN_n_(0, $\tau^{-1}I_{\text{n}}$); λ specifies the ratio between two variance components; τ^−1^ signifies the variance of the residual errors; *K* represents a genomic relatedness matrix between individuals was estimated via GEMMA; *I* refers to an n × n identity matrix, and n is the number of individuals; MVN_n_ is the n-dimensional multivariate normal distribution.

Furthermore, GCTA was used to estimate SNP-based genetic correlations between the three traits and phenotypic variance explained by the significant SNPs in the two Duroc pig populations.

### Multi-Trait GWAS

To test the multi-trait association between each SNP and the three traits in this study, GEMMA v0.98 was used to implement a multivariate linear mixed model (mvLMM). The mvLMM for the approach used in this study was previously studies described (18, 19). The LMM and mvLMM used the same covariates in this study. Notably, single-trait GWAS and multi-trait GWAS of two Duroc pig populations applied the same LMM and mvLMM in this study, respectively. Moreover, in the single-trait GWAS and multi-trait GWAS analysis, the genome-wide significant (0.05/N) and suggestive (1/N) thresholds via Bonferroni correction, in which N is the number of SNPs used in the analysis.

### Haplotype Block Analysis and Conditional Analysis

Haplotype block analysis was implemented for chromosomal regions with multiple significant SNPs using PLINK v1.9 and Haploview v4.2 (20). Moreover, we performed conditional analysis to detect the independence of all significant signals in the putative region. This analysis was conducted using the LMM by GEMMA software and added the top SNP genotypes as covariates into the LMM.

### Estimation of Genetic Parameters and the Explained Phenotypic Variance

#### Phenotypic Correlation

In this study, we calculated the phenotypic correlation (*r*_*p*_) of the sample *via* Pearson's correlation coefficient, and the formula for calculating the Pearson's correlation coefficient is given by (21).


$$r_{p}=\frac{\Sigma_{i=1}^{n}\left(X_{i}-\bar{X}\right)\left(Y_{i}-\bar{Y}\right)}{\sqrt{\Sigma_{i=1}^{n}\left(X_{i}-\bar{X}\right)}\;\sqrt[{2}]{\sum_{i=1}^{n}{\left(Y_{i}-\bar{Y}\right)}^{2}}}$$

where *X* and *Y* refer to the two traits; *r*_*p*_ is Pearson's correlation coefficient between the two traits; *n* represents the number of samples; *X*_*i*_ and *Y*_*i*_ are the phenotype values of *X* and *Y* traits for the i-th individual, respectively; $\bar{X}\;$and $\bar{Y}$ refer to the average phenotype value of the *X* and *Y* traits across all samples, respectively.

#### Genetic Correlation

GCTA software was used to estimate the genetic correlation between two traits by performing a bivariate genome-based restricted maximum likelihood (GREML) analysis. The formula for calculating the genetic correlation coefficient is given by (15, 22, 23).


$$\begin{array}{l} r_{g}=\frac{\sigma_{g1g2}}{\sigma_{g1}\sigma_{g2}} \end{array}$$

where *r*_*g*_ is genetic correlation coefficient between two traits; the subscripts “1” and “2” represent the two traits; σ_*g*1*g*2_ refers to the genetic covariance; σ_*g*_ represents square root of the genetic variance for the trait (captured by all SNPs).

#### The Explained Phenotypic Variance by the Significant SNP

The restricted maximum likelihood (REML) method was used to estimate the phenotypic variance explained by the significant SNPs for CC, AC, and CC traits by GCTA software. The model for calculating the phenotypic variance explained by the significant SNPs is given by


$$y=\text{Xβ}+g+\varepsilon \text{ with var}(\text{y}){\text{=A}}_{\text{g}}\;\sigma_{\text{g}}^{2}\text{+}I\;\sigma_{\varepsilon }^{2}$$

where *y* is to the vector of phenotype value; β represents a vector of fixed effects, including the top five eigenvectors of PCA; *X* refers to an incidence matrix for β; *g* is the vector of the aggregate effects of all the qualified SNPs for the pigs within one population; *I* is the identity matrix; *A*_*g*_ represents the genetic relationship matrix; $\sigma_{g}^{2}$ corresponds to the additive genetic variance captured by either the genome-wide SNPs or the selected SNPs, and $\sigma_{\varepsilon }^{2}$ refers to the residual variance. GCTA was used to estimate the genomic heritability for the three conformation traits in the bivariate REML model.

### Pathway Analyses

Genes annotation was based on the physical location of the significant SNPs in the Ensembl annotation of the *Sus scrofa* 11.1 genome version (http://ensembl.org/Sus_scrofa/Info/Index). To further identify potential candidate genes, KOBAS 3.0 (http://kobas.cbi.pku.edu.cn/kobas3) was used to perform Kyoto Encyclopedia of Genes and Genomes (KEGG) and Gene Ontology (GO) analyses (24).

## Results

### Phenotype Statistics and Correlation Among the Traits

**Figure 2:**
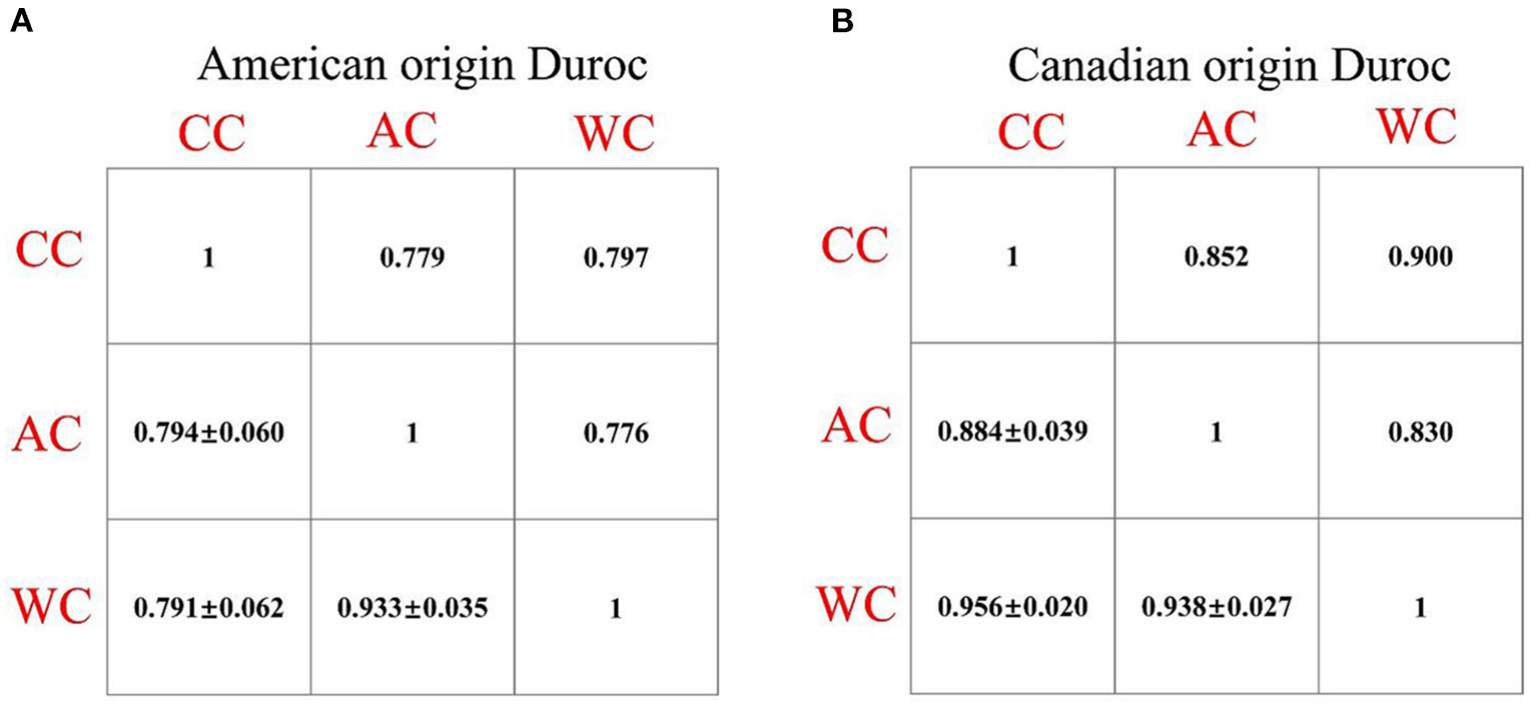
**(A,B)** Phenotypic (above diagonal) and genetic (below diagonal) correlations between the chest, abdominal, and waist circumferences traits in the two Duroc pig populations. In the plots, the below diagonal values show genetic correlations (standard error) between all the traits. All of the phenotypic correlation coefficients are significant with *P* < 0.05. CC, chest circumference; AC, abdominal circumference; WC, Waist circumference.

The statistical distribution and heritability of the traits measured in this study are presented in Table 1. In the AOD and COD pigs, the genomic heritabilities of the CC, AC, and WC traits were low, ranging from 0.11 to 0.15 (Table 1). Moreover, the coefficients of variation for CC, AC, and WC in the AOD and COD populations ranged from 3.13% to 3.33% and 3.91% to 4.06%, respectively (Table 1). The results showed that the phenotypic variations of the three traits in the COD population were higher than in the AOD population. Further demonstrated that the uniformities of these three conformation traits in AOD pigs were higher than that of COD pigs. Interestingly, our previous study showed that compared to the COD population, the coefficient of variation for the lean meat percentage (LMP) and average daily gain (ADG) was smaller in the AOD population (25). In addition, compared to the COD population, the mean of LMP and ADG were higher in the AOD population. Admittedly, the conformation traits can reflect the growth and physiological status of pigs. Thus, we considered that the uniformities of these three conformation traits in AOD pigs were higher, which might be because AOD pigs have a higher selection intensity for production traits than COD pigs. Moreover, genetic and phenotypic correlation coefficients among CC, AC, and WC traits were showed in Figure 2. High genetic correlation (*r* > 0.79) and phenotypic correlation coefficients (*r* > 0.77) were identified between all traits in the two Duroc pig populations. Given the high genetic correlation and phenotypic correlation coefficients between CC, AC, and WC traits, these traits may be improved together in pig breeding.

**Table 1:** Phenotype and heritability statistics for CC, AC, and WC.

**Population^a^**	**Traits^b^**	**N^c^**	**Mean (SD)^d^**	**C.V. (%)^e^**	**h^2^ (SE)^f^**
AOD	CC	2,206	106.73 ± 3.38 (cm)	3.17	0.12 ± 0.03
	AC		116.53 ± 3.64 (cm)	3.13	0.12 ± 0.04
	WC		104.50 ± 3.49 (cm)	3.33	0.11 ± 0.02
COD	CC	2,082	108.23 ± 4.23 (cm)	3.91	0.12 ± 0.03
	AC		115.77 ± 4.70 (cm)	4.06	0.15 ± 0.03
	WC		105.11 ± 4.22 (cm)	4.01	0.14 ± 0.03

a *American origin Duroc pig population (AOD), Canadian origin Duroc pig population (COD)*

b *Chest circumference (CC), abdominal circumference (AC), and waist circumference (WC)*.

c *Number (N)*.

d *Mean (standard deviation)*.

e *Coefficient of variation (C.V.)*.

f *Heritability (standard error)*.

### Population Stratification Assessment

As is known, population stratification can cause false-positive findings in GWAS analysis (26). Thus, PCA was conducted for experimental populations before GWAS analysis to detect the potential population stratification. The PCA plot was presented in our previous paper (25). The PCA plot showed that AOD and COD pigs were classified clearly into two clusters, indicating that these two populations have different genetic backgrounds. Thus, the two Duroc pig populations were analyzed separately. Moreover, Q-Q plots were also conducted for CC, AC, and WC in two Duroc populations to assess population stratification. The *P*-values of Q-Q plots indicated that no overall systematic biases were observed, and the genomic inflation factor (λ) at each trait amounted to 0.977 to 1.04, indicating that there was no population stratification (Supplementary Figure 1).

### Single-Trait GWAS

**Figure 3:**
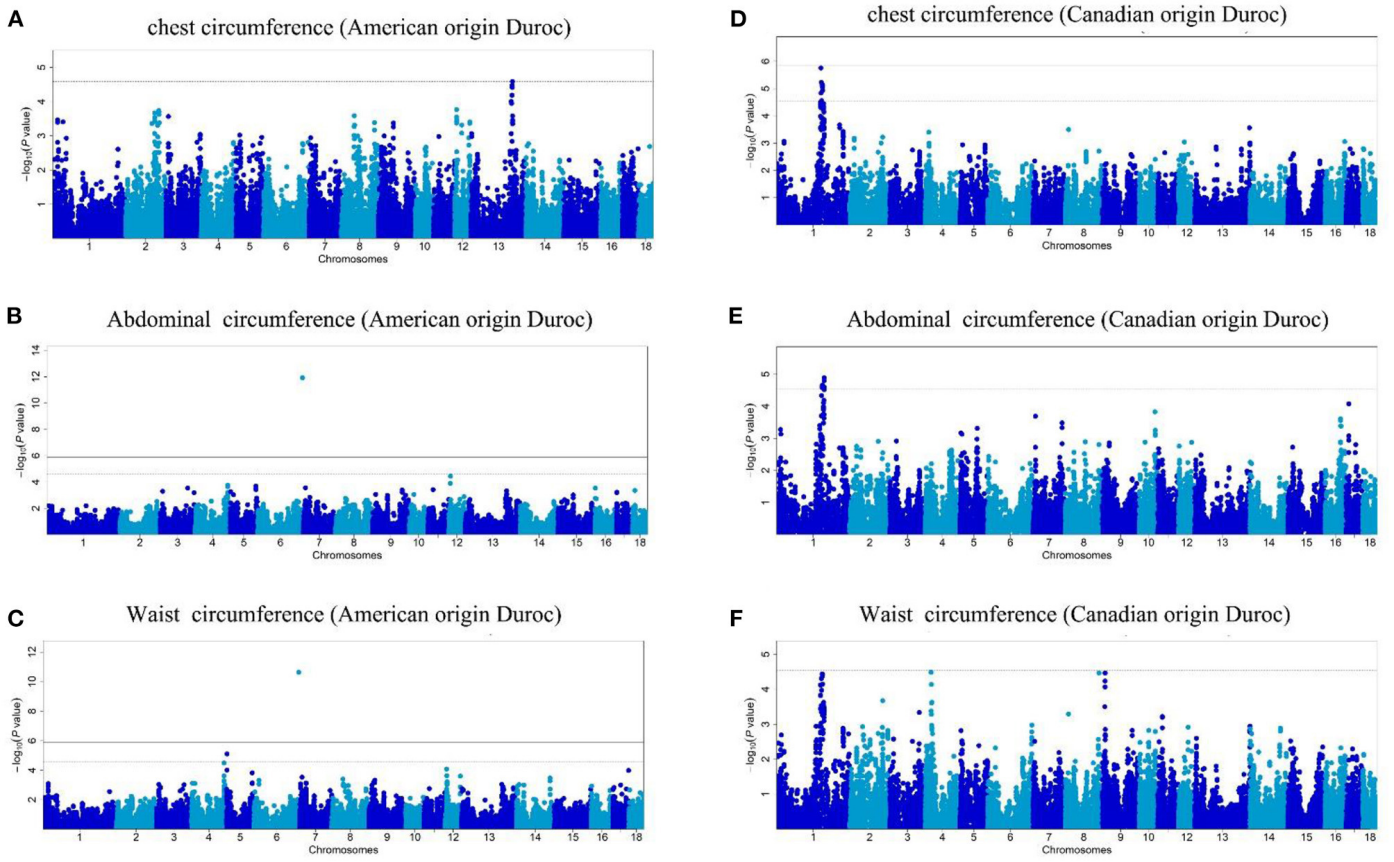
**(A–F)** Manhattan plots of single-trait GWAS for chest, abdominal, and waist circumferences in the two Duroc pig populations. In the Manhattan plots, the negative log10 *P*-values of the quantified SNPs were plotted against their genomic positions. Different blue colors denote various chromosomes. The solid and dashed lines represent the 5% genome-wide and chromosome-wide (suggestive) Bonferroni-corrected thresholds, respectively.

**Figure 4:**
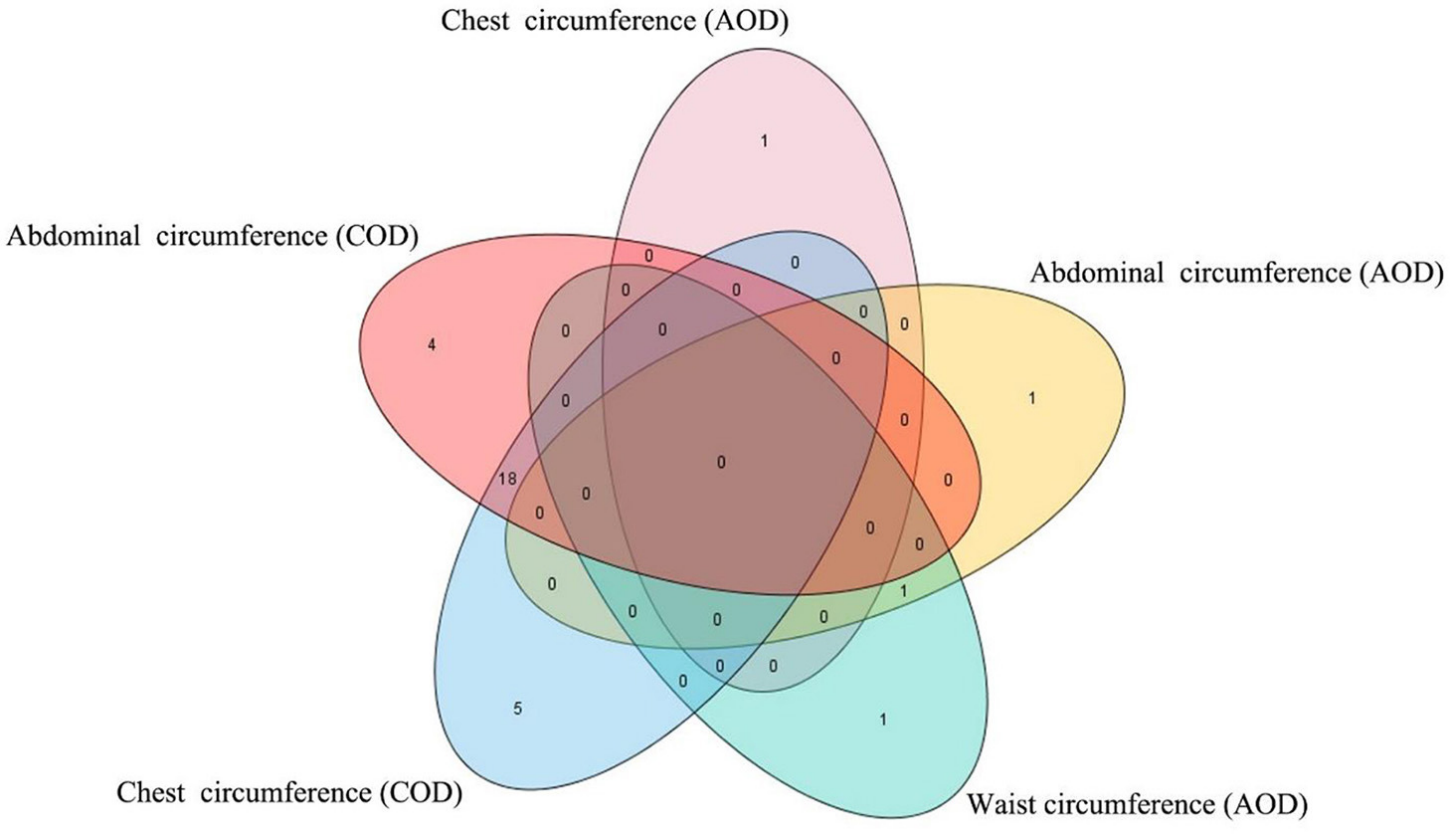
Venn plot of the significant SNPs detected by single-trait GWAS. The Venn plot was generated by software TBtools (27). AOD, American origin Duroc; COD, Canadian origin Duroc.

In this study, the AOD and COD pigs have different genetic backgrounds detected by PCA. Thus, the single-trait GWAS was implemented for the three conformation traits in the two Duroc populations, respectively. The single-trait GWAS results of the three conformation traits were presented in Figure 3 and Table 2. For the AOD pigs, single-trait GWAS identified one, two, and two SNPs were associated with CC, AC, and WC, respectively (Table 2). Among these significant SNPs, one genome-wide (*P* <1.40 × 10^−6^) SNP was associated with AC and WC (Table 2). The remaining three SNPs reached suggestive significant levels (*P* <2.80 × 10^−5^) (Table 2). For COD pigs, 23 and 22 suggestive SNPs were detected to be associated with CC and AC, respectively (Figure 3 and Table 2). However, no significant SNPs were identified to be associated with WC in COD pigs (Figure 3F). Notably, 19 pleiotropic SNPs were identified in this study. In these pleiotropic SNPs, one SNP on *Sus scrofa* chromosome (SSC) six was associated with AC and WC in the AOD pigs, and 18 SNPs were associated with CC and AC in the COD pigs (Figure 3 and Table 2). Interestingly, no common significant SNPs among the two Duroc pig populations were identified in this study, indicating the complex genetic architecture of CC, AC, and WC traits in pigs (Table 2 and Figure 4).

**Table 2:** Significant SNPs and candidate genes for CC, AC, and WC in the single-trait GWAS.

**Trait^a^ (population)**	**SNP^b^**	**SSC^c^**	**Location^d^ (bp)**	**EPV^e^ (%)**	***P*-value^f^**	**Distance^g^ (bp)**	**Candidate gene**
CC (AOD)	ASGA0059219	13	156576634	0.46	2.57 ×10^−5^	64,649	*ZPLD1*
AC (AOD)	**Affx-114594216**	6	168268278	0.28	**1.18** **×10**^**−12**^	13,851	*ENSSSCG00000039458*
	WU_10.2_12_6509723	12	6520803	0.45	4.49 ×10^−6^	−4,504	*ENSSSCG00000037541*
WC (AOD)	ASGA0104919	5	168268278	0.91	7.84 ×10^−6^	248,426	*TBC1D22A*
	**Affx-114594216**	6	168268278	0.31	**2.26** **×10**^**−11**^	64,649	*ZPLD1*
CC (COD)	ALGA0006817	1	166363826	0.02	1.75 ×10^−6^	−52,854	*ITGA11*
	ALGA0006891	1	167622960	0.22	5.90 ×10^−6^	158,549	*TLE3*
	ALGA0006895	1	167690373	0.22	5.90 ×10^−6^	118,136	*TLE3*
	**ALGA0006973**	1	170618790	0.34	6.76 ×10^−6^	−479,665	*ENSSSCG00000041595*
	**WU_10.2_1_189388692**	1	170473535	0.33	7.75 ×10^−6^	−334,410	*ENSSSCG00000041595*
	**ALGA0006975**	1	170605277	0.33	7.75 ×10^−6^	−466,152	*ENSSSCG00000041595*
	**ALGA0006977**	1	170675822	0.33	7.75 ×10^−6^	−536,697	*ENSSSCG00000041595*
	**ALGA0006982**	1	170979266	0.33	7.75 ×10^−6^	−840,141	*ENSSSCG00000041595*
	**MARC0033388**	1	171017646	0.33	7.75 ×10^−6^	−878,521	*ENSSSCG00000041595*
	**ALGA0006991**	1	171052112	0.33	7.75 ×10^−6^	854,086	*LRFN5*
	**ALGA0006996**	1	171120240	0.33	7.75 ×10^−6^	785,958	*LRFN5*
	**DRGA0001638**	1	171166693	0.33	7.75 ×10^−6^	739,505	*LRFN5*
	**DRGA0001642**	1	171311843	0.33	7.75 ×10^−6^	594,355	*LRFN5*
	**ALGA0007002**	1	171545925	0.33	7.75 ×10^−6^	360,273	*LRFN5*
	**Affx-114729299**	1	171652441	0.33	7.75 ×10^−6^	253757	*LRFN5*
	**MARC0033468**	1	172184742	0.33	7.75 ×10^−6^	−6,792	*LRFN5*
	**MARC0002276**	1	172219301	0.33	7.75 ×10^−6^	−41,351	*LRFN5*
	**MARC0080275**	1	172136167	0.32	8.87 ×10^−6^	within	*LRFN5*
	**ASGA0005303**	1	172156509	0.32	8.87 ×10^−6^	within	*LRFN5*
	**WU_10.2_1_192315864**	1	172806698	0.33	1.16 ×10^−5^	−628,748	*LRFN5*
	**ALGA0007021**	1	172988615	0.33	1.16 ×10^−5^	−810,665	*LRFN5*
	MARC0053979	1	166608531	0.27	1.45 ×10^−5^	−146	*CORO2B*
	ASGA0005260	1	168817608	0.24	2.76 ×10^−5^	−17,083	*ENSSSCG00000045715*
AC (COD)	MARC0054010	1	178298953	0.30	1.28 ×10^−5^	−705,276	*MDGA2*
	DRGA0001741	1	178372667	0.31	1.59 ×10^−5^	−778,990	*MDGA2*
	**MARC0080275**	1	172136167	0.42	2.17 ×10^−5^	within	*LRFN5*
	**ASGA0005303**	1	172156509	0.42	2.17 ×10^−5^	within	*LRFN5*
	**WU_10.2_1_189388692**	1	170473535	0.42	2.30 ×10^−5^	−334,410	*ENSSSCG00000041595*
	**ALGA0006975**	1	170605277	0.42	2.30 ×10^−5^	−466,152	*ENSSSCG00000041595*
	**ALGA0006977**	1	170675822	0.42	2.30 ×10^−5^	−536,697	*ENSSSCG00000041595*
	**ALGA0006982**	1	170979266	0.42	2.30 ×10^−5^	−840,141	*ENSSSCG00000041595*
	**MARC0033388**	1	171017646	0.42	2.30 ×10^−5^	−878,521	*ENSSSCG00000041595*
	**ALGA0006991**	1	171052112	0.42	2.30 ×10^−5^	854,086	*LRFN5*
	**ALGA0006996**	1	171120240	0.42	2.30 ×10^−5^	785,958	*LRFN5*
	**DRGA0001638**	1	171166693	0.42	2.30 ×10^−5^	739,505	*LRFN5*
	**DRGA0001642**	1	171311843	0.42	2.30 ×10^−5^	594,355	*LRFN5*
	**ALGA0007002**	1	171545925	0.42	2.30 ×10^−5^	360,273	*LRFN5*
	**Affx-114729299**	1	171652441	0.42	2.30 ×10^−5^	253,757	*LRFN5*
	**MARC0033468**	1	172184742	0.42	2.30 ×10^−5^	−6,792	*LRFN5*
	**MARC0002276**	1	172219301	0.42	2.30 ×10^−5^	−41,351	*LRFN5*
	**WU_10.2_1_192315864**	1	172806698	0.43	2.43 ×10^−5^	−628,748	*LRFN5*
	**ALGA0007021**	1	172988615	0.43	2.43 ×10^−5^	−810,665	*LRFN5*
	WU_10.2_1_199274501	1	178975482	0.25	2.51 ×10^−5^	272,265	*ENSSSCG00000046948*
	WU_10.2_1_199687896	1	179301234	0.26	2.61 ×10^−5^	−47,692	*ENSSSCG00000046948*
	**ALGA0006973**	1	170618790	0.42	2.61 ×10^−5^	−479,665	*ENSSSCG00000041595*

a *CC (AOD), Chest circumference (American origin Duroc); AC (AOD), abdominal circumference (American origin Duroc); WC (AOD), waist circumference (American origin Duroc); CC (COD), Chest circumference (Canadian origin Duroc); AC (COD), abdominal circumference (Canadian origin Duroc)*.

b *SNP ID in boldface represents the SNP had pleiotropic effects on the conformation traits*.

c *SSC, Sus scrofa chromosome*.

d *SNP positions in Ensembl*.

e *EPV, Explained phenotypic variance*.

f *P-value in boldface: genome-wide significant; P-value not in boldface: suggestive significant*.

g *+/: the SNP located upstream/downstream of the nearest gene*.

### Haplotype Block Analysis

**Figure 5:**
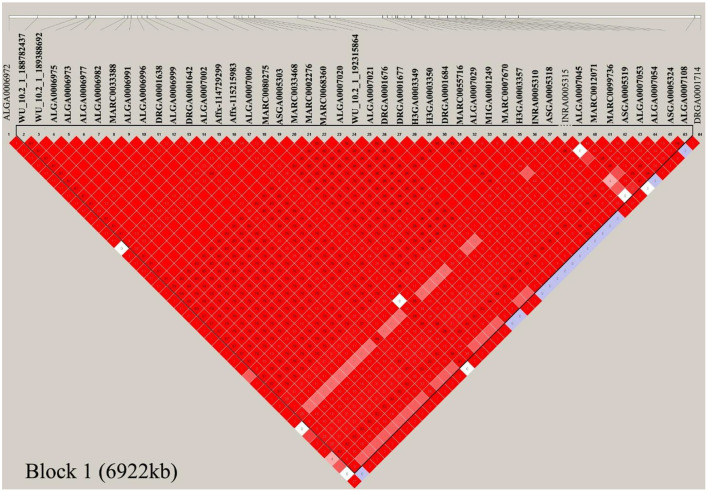
Haplotype blocks on SSC1 for chest and Abdominal circumference in the COD pigs. Haplotype blocks are marked with triangles. Values in boxes are the linkage disequilibrium (*r*^2^) between the SNP pairs. The haplotype blocks are colored in accordance with the standard Haploview color scheme: LOD > 2 and D' = 1, red; LOD <2 and D' = 1, blue; LOD <2 and D' <1, white (LOD is the log of the likelihood odds ratio, a measure of confidence in the value of D').

**Figure 6:**
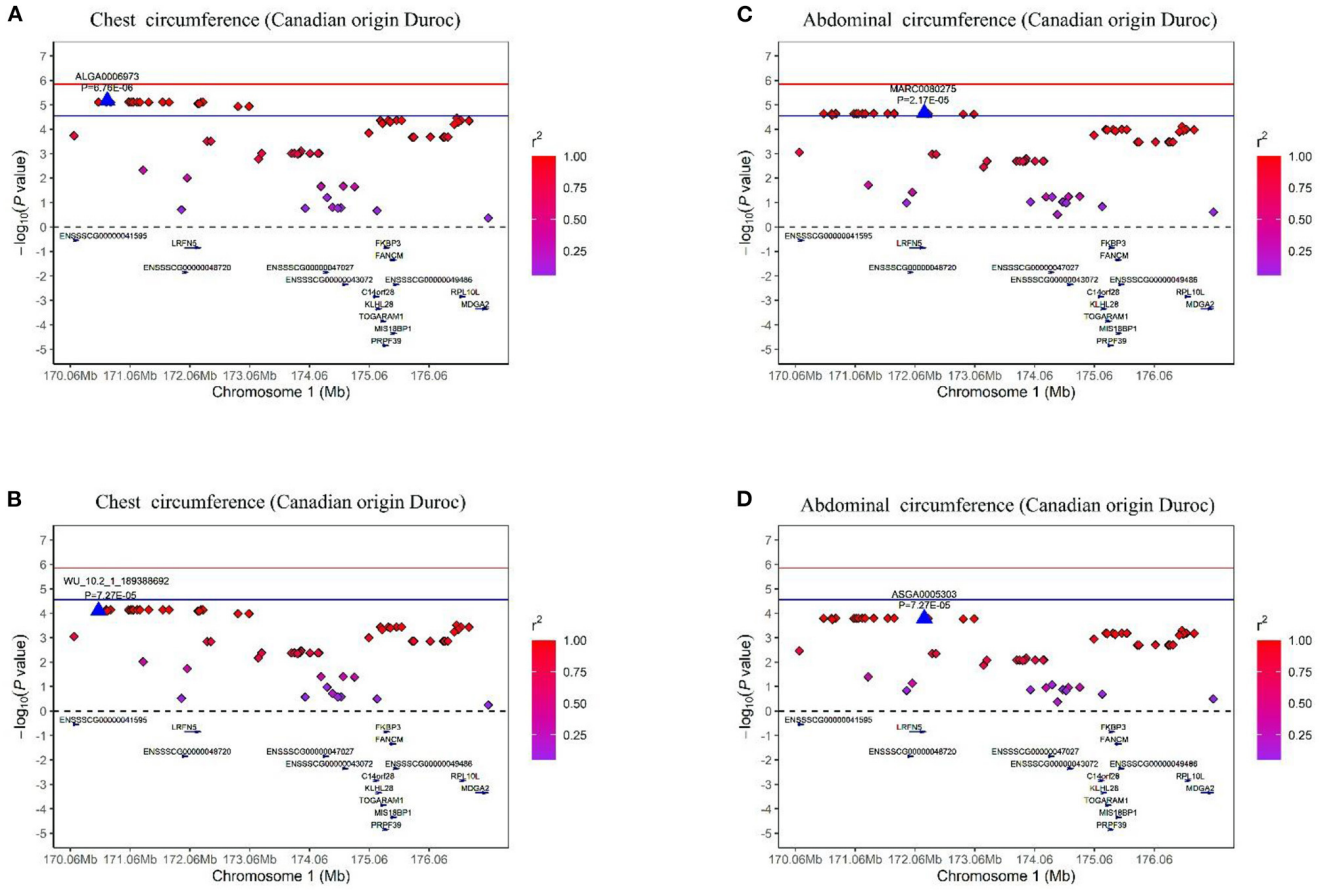
Regional association plots of the top SNP ALGA0006973 and MARC0080275 were associated with the chest and abdominal circumferences at SSC1, respectively. In the plots, the red and blue represent the 5% genome-wide and chromosome-wide (suggestive) Bonferroni-corrected thresholds, respectively. The significant SNP are indicated by big blue triangles. SNPs are denoted by colored diamonds depending on the target SNP with which they were in strongest LD. The plots indicate the association results for CC on the COD pigs **(A)** before and **(B)** after conditional analysis on ALGA0006973. The plots indicate the association results for AC on the COD pigs **(C)** before and **(D)** after conditional analysis on MARC0080275.

In this study, multiple SNPs were in close proximity to each other and were detected to be associated with the same traits. Notably, 18 SNPs on SSC1 were identified to be associated with the CC and AC traits in the COD pigs and were situated in a haplotype block between 170.06 and 176.98 Mb (6.92 Mb) (Table 2 and Figure 5). These results are not surprising because of the high genetic correlation (*r* = 0.884) and phenotypic correlation coefficients (*r* = 0.852) between CC and AC in the COD pigs (Figure 2). In the QTL region, the ALGA0006973 and MARC0080275 were the most significant SNP for CC and AC, respectively. The top SNPs ALGA0006973 and MARC0080275 for this QTL region explained 0.34% and 0.42% of the phenotypic variance for CC and AC, respectively (Table 2). To examine whether linkage disequilibrium (LD) caused the associations, we conducted conditional analyses for the CC and AC. Then, the top SNP ALGA0006973 for CC and the top SNP MARC0080275 for AC were fitted into the LMM as a covariate to conduct conditional GWAS, respectively. For the CC trait, many SNPs in high LD with the top SNP were significant in the single-trait GWAS (Figure 6A), but the *P*-values of these significant SNPs decreased below the minimum threshold line after the top SNP ALGA0006973 was included as a fixed effect in the model (Figure 6B). The same pattern was observed for the top SNP MARC0080275 for the AC trait (Figures 6C,D). These results indicated that the QTL on SSC1 might have pleiotropic effects on CC and AC in COD pigs, which spans 6.92 Mb (from 170.06 to 176.98 Mb).

### Multi-Trait GWAS Results

In this study, we performed multi-triat GWASs for the CC, AC, and WC traits in the two Duroc pig populations, respectively. A total of 21 significant SNPs have pleiotropic effects on CC, AC, and WC by multi-trait GWAS detected. For the AOD pigs, multi-trait GWAS detected two SNPs were associated with the correlated traits, of which one SNP reached genome-wide significant levels (Supplementary Table 2 and Supplementary Figure 2). Furthermore, compared to single-trait GWAS, multi-trait GWAS detected one additional SNP in the AOD pigs (Supplementary Table 2 and Supplementary Figure 3A). For the COD pigs, multi-trait GWAS identified 19 suggestive SNPs associated with these correlated traits, of which three SNPs were undetected in the three single-traits GWASs (Supplementary Table 2 and Supplementary Figure 3B). These results showed that multi-trait GWAS detected additional pleiotropic SNPs and confirmed most of the significant SNPs in the single-trait GWAS, suggesting it can increase statistical power and complement single-trait GWAS results.

### Candidate Genes and Functional Annotation

Amounted to eight candidate genes located within or near the significant SNPs that were associated with the three conformation traits (Table 2 and Supplementary Table 2). To further explore the eight candidate genes involved in pathways and biological processes, these candidate genes were used to conduct KEGG pathways and GO analysis. The KEGG pathways and GO analysis indicated that the candidate genes for the three conformation traits mainly participated in sphingolipid metabolism and lysosome pathways (Supplementary Table 3).

## Discussion

### Comparing Single-Trait GWAS With Multi-Trait GWAS

Given that the two Duroc pig populations have different genetic backgrounds, the single-trait GWASs were performed for the CC, AC, and WC traits in the two Duroc populations, respectively. In addition, we found that high phenotype and genetic correlation exist between CC, AC, and WC in the two Duroc pig populations (Figure 2). Multi-trait GWAS is usually used to detect QTLs that were associated with multiple traits. The higher the genetic and phenotypic correlation between traits, the higher the statistical power of the multi-trait GWAS (28). Thus, to improve the power of GWAS detection, we conducted multi-trait GWAS for these three correlated traits in the two Duroc pig populations, respectively. The single-trait GWAS detected 31 SNPs were associated with the conformation traits, of which 19 SNPs may have pleiotropic effects on the conformation traits. The multi-trait GWAS identified 21 SNPs that were associated with the three conformation traits (Table 2). Compared to single-trait GWAS, multi-trait GWAS detected four additional SNPs (Supplementary Figure 3 and Supplementary Table 2). Many previous studies demonstrated that the combination strategy of single-trait and multi-trait GWAS could improve the power of GWAS. Chhetri et al. (29) conducted single-trait and multi-trait GWASs for morphological and physiological traits in Populus trichocarpa trees. A total of four and 20 gene models were identified by the single-trait and multi-trait GWASs, respectively. Yan et al. (19) performed single-trait and multi-trait GWASs for hematological traits in the White Duroc × Erhualian F_2_ resource population. The results showed that compared to single-trait GWAS, multi-trait GWAS detected a total of 16 newly significant loci for the hematological traits. These results showed that multi-trait GWAS could complement the single-trait GWAS results to increase the statistical power of GWAS.

Interestingly, no common significant SNPs among the two Duroc pig populations were identified in this study, indicating the complex genetic architecture of CC, AC, and WC traits in pigs (Figure 4 and Supplementary Table 2). Our results were consistent with many previous studies. For example, Tang et al. (30) conducted three GWASs for teat number in the Erhualian, Sutai, and F_2_ (White Duroc × Erhualian) pigs, and none of the loci was shared by the three pig populations or two of these populations. Bergfelder et al. (31) performed GWAS for the number of piglets born alive on the Large White and Landrace pigs, and no common significant SNP or QTL region between Large White and Landrace breed were detected. These differences in GWAS results may be caused by differences in the minor allele frequency of SNP in different breeds or populations belonging to the same breed, which further reflected the genetic backgrounds that could greatly affect the single-marker associations.

### Comparison With Previously Mapped QTL in Pigs

In the study, we performed single-trait and multi-trait GWASs for CC, AC, and WC in 2,206 AOD and 2,082 COD pigs. A total of 35 significant SNPs on SSC1, SSC4, SSC5, SSC6, SSC7, SSC12, and SSC13 were identified in this study. According to the significant SNP and QTL positions in this study, evaluate whether these SNPs and QTLs are located in the previously reported QTLs from the pigQTLdb. However, none of the significant SNPs in this study are observed to be included in any previously reported QTLs that are associated with CC, AC, and WC in pigs. This difference might be due to the difference in the genotypes and the breeds used in this study or few studies for CC, AC, and WC in pigs. In brief, in this study, a total of 35 significant SNPs were newly detected to be associated with CC, AC, and WC in pigs, which may be useful for marker-assisted selection in pig breeding.

### Candidate Genes and Functional Annotation

According to the results of the bioinformatics analysis and functions of candidate genes, three genes (Integrin Subunit Alpha 11 [*ITGA11*], TLE Family Member 3, Transcriptional Corepressor [*TLE3*], and Galactosylceramidase [*GALC*]) associated with the conformation traits were selected candidates. The *ITGA11* gene is near SNP ALGA0006817, and the SNP was identified to be associated with CC. Popova et al. (32) found that *ITGA11* knockout mice display a smaller skeletal system than wild-type mice. Moreover, the *ITGA11* gene was reported to be involved in maintaining adult skeletal bone mass (33). This indicated that *ITGA11* might be an essential regulator of skeletal bone mass, so it should be considered a strong candidate gene for CC. Two identified SNPs (ALGA0006891 and ALGA0006895) on SSC1 were associated with CC, and the SNPs are located nearest the *TLE3* gene. The *TLE3* was identified as a dual function modulator of adipogenesis that augments PPARγ action and inhibits Wnt signaling, and PPARγ and Wnt signaling are central positive and negative regulators of adipogenesis, respectively (34). Three significant SNPs (MARC0087724, WU_10.2_7_116331723, and H3GA0022932) on SSC7 are located nearest or within the *GALC* gene. These SNPs were detected to be associated with the CC, AC, and WC traits. The *GALC* gene encodes a lysosomal protein (35). Many studies found that lysosomes participated in regulating lipid metabolism (36, 37). Interestingly, the bioinformatics analysis showed that the *GALC* is mainly involved in sphingolipid metabolism and lysosome pathways (Supplementary Table 3). The lysosomes were reported to be involved in the degradation of sphingolipids (38). Sphingolipid production can lead to lipid accumulation disorders (39). These results suggested that the *TLE3* and *GALC* genes may play an important role in lipid metabolism. As we all know, fat is the main factor affecting body size, so the *TLE3* and *GALC* genes should be regarded as strong candidate genes for the conformation traits.

## Conclusion

This study conducted single-trait and multi-trait GWASs for CC, AC, and WC in 2,206 AOD and 2,082 COD pigs. As a result, we detected a total of 35 newly significant SNPs for CC, AC, and WC on SSC1, SSC4, SSC5, SSC6, SSC7, SSC12, and SSC13. Among these SNPs, single-trait GWAS detected 18 significant SNPs on SSC1 were associated with CC and AC in COD pigs. These 18 SNPs were situated in a QTL region with a 6.92 Mb interval (from 170.06 to 176.98 Mb). Moreover, 21 newly pleiotropic SNPs were identified to be associated with the three conformation traits by multi-trait GWAS, suggesting the multi-trait GWAS is a powerful statistical method to identify pleiotropic locus. According to the bioinformatics analysis and the functions of candidate genes, three genes (*ITGA11, TLE3*, and *GALC*) may affect skeletal bone mass and lipid metabolism. Finally, three genes were selected as strong candidate genes that may affect the conformation traits. Our findings further reveal the genetic architecture of CC, WC, and AC, which may provide new insights for improving the conformation traits in pigs.

## Data Availability Statement

The datasets presented in this study can be found in online repositories. The names of the repository/repositories and accession number(s) can be found at: https://figshare.com/, 1.

## Ethics Statement

The animal study was reviewed and approved by the Animal Care and Use Committee of the South China Agricultural University (SCAU) (Guangzhou, China).

## Author Contributions

JY, MY, and ZW conceived and designed the experiments and revised the manuscript. SZ, RD, ZZ, HZ, SW, DR, JW, YQ, and EZ collected the samples, recorded the phenotypes, and performed the experiments. SZ and RD analyzed the data. ZW and GC provided the material. SZ, RD, and MY wrote the manuscript. All authors contributed to the article and approved the submitted version.

## Funding

This study was supported by the Guangdong Yang Fan Innovative and Entrepreneurial Research Team Program (2016YT03H062) and the National and Provincial Fund Cultivation Project of Zhongkai College of Agricultural Engineering.

## Conflict of Interest

RD, HZ, SW, GC, and ZW were employed by company Guangdong Wens Breeding Swine Technology Co., Ltd. The remaining authors declare that the research was conducted in the absence of any commercial or financial relationships that could be construed as a potential conflict of interest.

## Publisher's Note

All claims expressed in this article are solely those of the authors and do not necessarily represent those of their affiliated organizations, or those of the publisher, the editors and the reviewers. Any product that may be evaluated in this article, or claim that may be made by its manufacturer, is not guaranteed or endorsed by the publisher.
