# Supplementary material for: Genome-Wide Association Analysis Reveals Genetic Loci and Candidate Genes for Chest, Abdominal, and Waist Circumferences in Two Duroc Pig Populations
Source: Front Vet Sci. 2022 Jan 28;8:807003. doi: 10.3389/fvets.2021.807003 (PMC8865076; doi:10.3389/fvets.2021.807003)
Supplement: Supplementary file 1 [file Data_Sheet_1.DOCX]

***Supplementary Material***

**
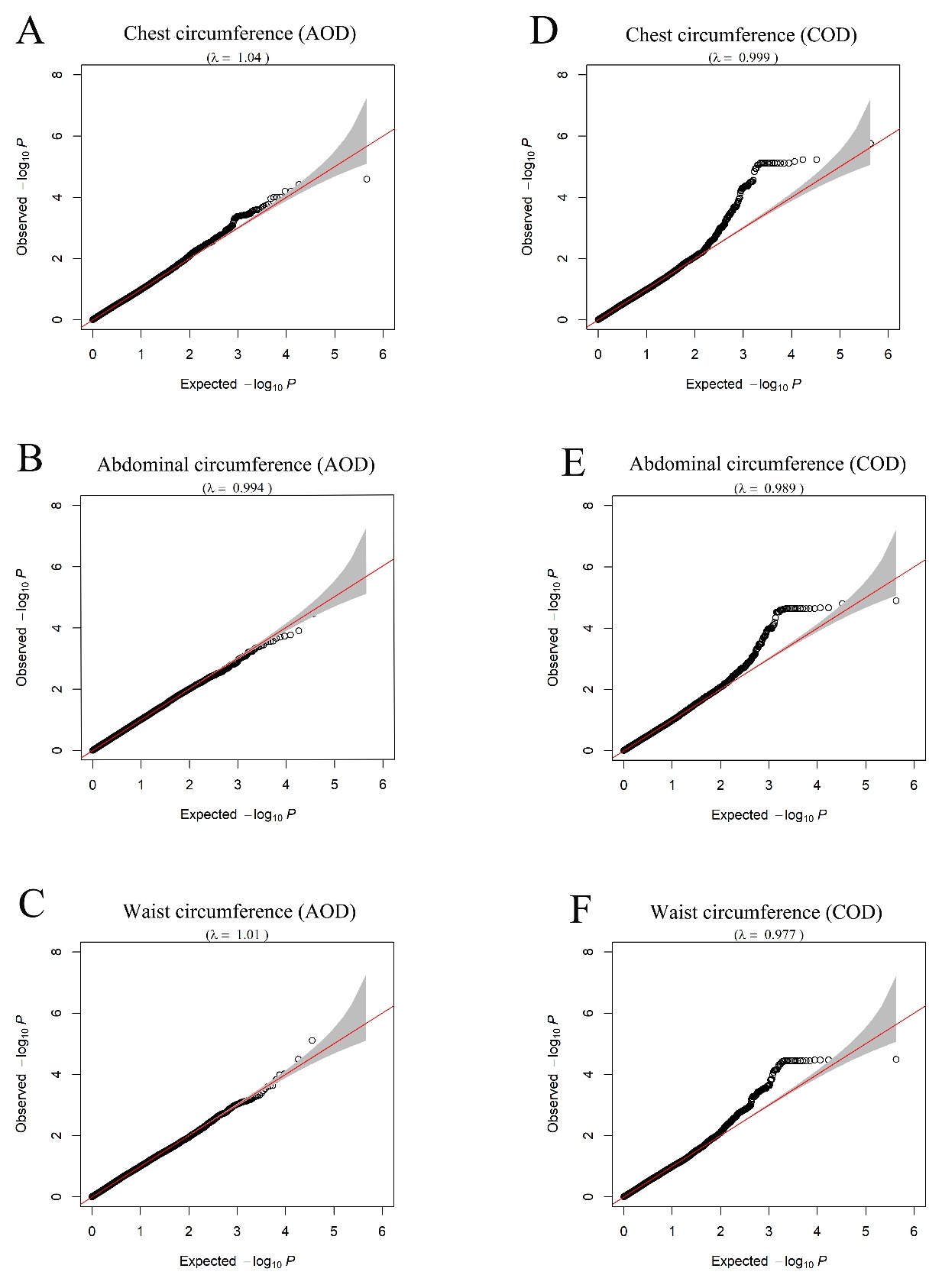
**

**Supplementary Figure 1.** Q–Q plots showing the observed versus expected log *P*-values for chest circumference, abdominal circumference, and waist circumference. The estimated lambda(λ) is shown in the figure. AOD: American origin Duroc; COD: Canadian origin Duroc.


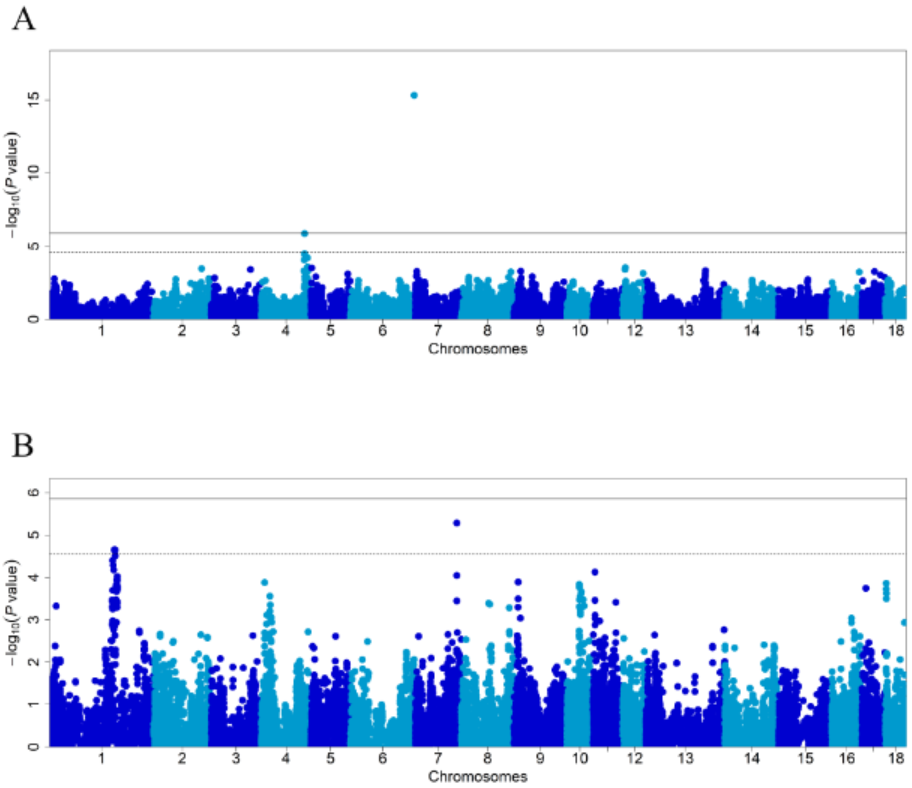


**Supplementary Figure 2.** Manhattan plots of multi-trait GWAS for results for chest circumference, abdominal circumference, and waist circumference in the Duroc pig populations. In the Manhattan plots, the solid and dashed lines represent the 5% genome-wide and chromosome-wide (suggestive) Bonferroni-corrected thresholds, respectively. Manhattan plot for **(A)** chest circumference, abdominal circumference, and waist circumference (American origin Duroc), **(B)** chest circumference, abdominal circumference, and waist circumference (Canadian origin Duroc).

**
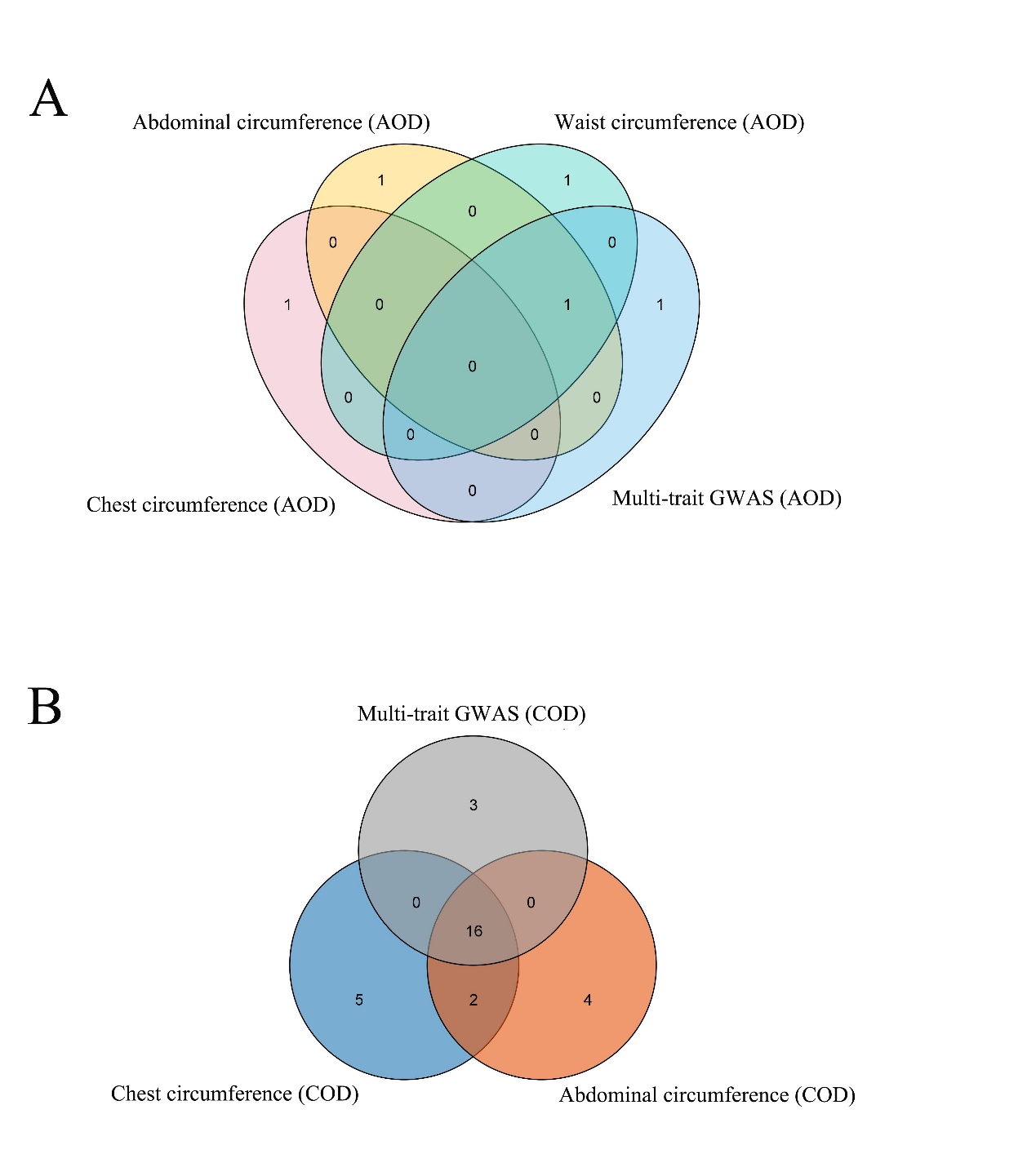
**

**Supplementary Figure 3.** Venn plot showing relationships of the significant SNPs that were identified by single-trait and multi-trait GWASs. AOD: American origin Duroc; COD: Canadian origin Duroc.

**Supplementary Table 1.** Effective SNPs for chest circumference, abdominal circumference, and waist circumference traits.

| Method | Traits | Population^1^ | SNP^2^ | | N^3^ |
| --- | --- | --- | --- | --- | --- |
| Single-trait GWAS | Chest circumference | AOD | 38592 | 2206 | |
|  | Abdominal circumference | AOD | 38592 | 2206 | |
|  | Waist circumference | AOD | 38592 | 2206 | |
|  | Chest circumference | COD | 35755 | 2082 | |
|  | Abdominal circumference | COD | 35755 | 2082 | |
|  | Waist circumference | COD | 35755 | 2082 | |
| Multi-trait GWAS | All the traits | AOD | 38592 | 2206 | |
|  | All the traits | COD | 35755 | 2082 | |

^1^AOD: American origin Duroc; COD: Canadian origin Duroc.

^2^The number of SNPs used in association analysis.

^3^N is number of pigs used in the analysis.

**Supplementary Table 2.** Significant SNPs and candidate genes for chest circumference, abdominal circumference, and waist circumference in multi-traits GWAS.

| Population^1^ | SSC^2^ | SNP^3^ | Location^4^  (bp) | *P-*value^5^ | Distance^6^  (bp) | Candidate gene |
| --- | --- | --- | --- | --- | --- | --- |
| AOD | 4 | **WU_10.2_4_125260677** | 114290317 | 1.35 × 10^-6^ | 646272 | *ENSSSCG00000035697* |
|  | 6 | Affx-114594216 | 168268278 | **4.63 × 10^-16^** | 13851 | *ENSSSCG00000039458* |
| COD | 1 | WU_10.2_1_189388692 | 170473535 | 2.20 × 10^-5^ | -334410 | *ENSSSCG00000041595* |
|  | 1 | ALGA0006975 | 170605277 | 2.20 × 10^-5^ | -466152 | *ENSSSCG00000041595* |
|  | 1 | ALGA0006977 | 170675822 | 2.20 × 10^-5^ | -536697 | *ENSSSCG00000041595* |
|  | 1 | ALGA0006982 | 170979266 | 2.20 × 10^-5^ | -840141 | *ENSSSCG00000041595* |
|  | 1 | MARC0033388 | 171017646 | 2.20 × 10^-5^ | -878521 | *ENSSSCG00000041595* |
|  | 1 | ALGA0006991 | 171052112 | 2.20 × 10^-5^ | 854086 | *LRFN5* |
|  | 1 | ALGA0006996 | 171120240 | 2.20 × 10^-5^ | 785958 | *LRFN5* |
|  | 1 | DRGA0001638 | 171166693 | 2.20 × 10^-5^ | 739505 | *LRFN5* |
|  | 1 | DRGA0001642 | 171311843 | 2.20 × 10^-5^ | 594355 | *LRFN5* |
|  | 1 | ALGA0007002 | 171545925 | 2.20 × 10^-5^ | 360273 | *LRFN5* |
|  | 1 | Affx-114729299 | 171652441 | 2.20 × 10^-5^ | 253757 | *LRFN5* |
|  | 1 | MARC0033468 | 172184742 | 2.20 × 10^-5^ | -6792 | *LRFN5* |
|  | 1 | MARC0002276 | 172219301 | 2.20 × 10^-5^ | -41351 | *LRFN5* |
|  | 1 | ALGA0006973 | 170618790 | 2.26 × 10^-5^ | -479665 | *ENSSSCG00000041595* |
|  | 1 | MARC0080275 | 172136167 | 2.29 × 10^-5^ | within | *LRFN5* |
|  | 1 | ASGA0005303 | 172156509 | 2.29 × 10^-6^ | within | *LRFN5* |
|  | 7 | **MARC0087724** | 109839696 | 5.20 × 10^-6^ | 54492 | *GALC* |
|  | 7 | **WU_10.2_7_116331723** | 109877373 | 5.20 × 10^-6^ | 16815 | *GALC* |
|  | 7 | **H3GA0022932** | 109963521 | 5.20 × 10^-6^ | within | *GALC* |

^1^AOD: American origin Duroc; COD: Canadian origin Duroc

^2^*Sus scrofa* chromosome (SSC).

^3^SNP ID in boldface: multi-trait GWAS identified the SNPs that were undetected in the single-traits GWASs.

^4^SNP positions in Ensembl.

^5^*P*-value in boldface: genome-wide significant; *P*-value in regular: suggestive significant.

^6^+/−: the SNP located upstream/downstream of the nearest gene.

**Supplementary Table 3.** KEGG PATHWAY and GO significant terms with the body size traits (*P* < 0.05).

| Term | Database | ID | *P*-Value | Gene names |
| --- | --- | --- | --- | --- |
| Sphingolipid metabolism | KEGG PATHWAY | ssc00600 | 0.012485905 | *GALC* |
| Arrhythmogenic right ventricular cardiomyopathy (ARVC) | KEGG PATHWAY | ssc05412 | 0.019707452 | *ITGA11* |
| ECM-receptor interaction | KEGG PATHWAY | ssc04512 | 0.022019029 | *ITGA11* |
| Hypertrophic cardiomyopathy (HCM) | KEGG PATHWAY | ssc05410 | 0.023301219 | *ITGA11* |
| Dilated cardiomyopathy (DCM) | KEGG PATHWAY | ssc05414 | 0.024325933 | *ITGA11* |
| Lysosome | KEGG PATHWAY | ssc04142 | 0.030709541 | *GALC* |
| Focal adhesion | KEGG PATHWAY | ssc04510 | 0.049896364 | *ITGA11* |
